# Commentary: Antibodies against multiple post-translationally modified proteins aid in diagnosis of autoimmune hepatitis and associate with complete biochemical response to treatment

**DOI:** 10.3389/fmed.2023.1275838

**Published:** 2023-09-21

**Authors:** Richard Taubert, Bastian Engel, Alejandro Campos-Murguia

**Affiliations:** Department of Gastroenterology, Hepatology, Infectious Diseases and Endocrinology, Hannover Medical School, Hannover, Germany

**Keywords:** autoimmune hepatitis (AIH), bovine serum albumin (BSA), fetal calf serum (FCS), immunoglobulin G (IgG), polyreactive IgG (pIgG), post-translational modification (PTM)

We read with great interest the article “*Antibodies against multiple post-translationally modified proteins aid in diagnosis of autoimmune hepatitis and associate with complete biochemical response to treatment*” by van den Beukel et al. (1). First of all, we would like to congratulate the authors on their interesting finding that immunoglobulin G (IgG) against post-translationally modified (PTM) proteins are also found in autoimmune liver diseases and especially in untreated autoimmune hepatitis (AIH) and not only in rheumatologic autoimmune diseases. In particular, the association of antibody patterns of positivity for multiple anti-PTM antibodies with treatment response has the potential to impact the care of patients with AIH.

Recently, we have identified a polyreactivity of IgG (pIgG) especially in patients with untreated AIH. That is, IgG from patients with untreated AIH were significantly more reactive against multiple foreign protein and non-protein antigens, as well as against multiple autoantigens, than patients with non-AIH liver diseases or healthy controls in adults and children (2, 3). We identified pIgG with a similar solid phase ELISA immunoassay using the same plastic ELISA plates (Nunc Maxisorb) with the same blocking reagent bovine serum albumin (BSA) as van den Beukel et al. (1). In addition, IgG from AIH patients showed increased binding to these BSA-blocked ELISA plates even without the addition of foreign antigens or autoantigens to the ELISA plates. Interestingly, BSA showed the strongest binding of pIgG of all blocking reagents tested. The addition of some (e.g., HIP1R) but not all autoantigens tested (e.g., ITSN1) further increased the binding of pIgG compared to BSA alone. This polyreactivity could only partially be eliminated by further dilution, pre-incubation of patient serum with the antigens of interest, ultracentrifugation to get rid of possible IgG immune complexes, or modification of the ELISA. The latter could include e.g., titration of different concentrations of NaCl and Tween as we did in our original publication for pIgG. The assay of van den Beukel et al. (1) contains similar ingredients.

Other parallels between the polyreactivity of IgG described by us and the anti-PTM antibodies described by van den Beukel et al. (1) are: I. PIgG and anti-PTM antibodies were mostly measurable in untreated AIH and concentrations of both pIgG and anti-PTM declined after initiation of immunosuppressive therapy. II. PIgG and anti-PTM antibodies had similar sensitivity in AIH being positive for conventional autoantibodies e.g., ANA and anti-SMA and in seronegative AIH. III. PIgG and anti-PTM antibodies exhibited comparable sensitivities and specificity for the differentiation of AIH from non-AIH liver diseases, as far as different cohorts are comparable.

While we used the autoantigen HIP1R identified in an autoantibody screening in a protein array (3), van den Beukel et al. (1) used modified and unmodified fecal calf serum (FCS), whose main protein component is BSA. It is our understanding that van den Beukel et al. (1) used ELISA with modified FCS, means mainly modified BSA, followed by BSA blocking. We wonder if the reactivity to anti-PTM FCS is different from the polyreactivity of IgG that we have recently identified. Have the authors compared the results of the anti-PTM assay with the pure reactivity in the same BSA-blocked ELISA, but without the addition of PTM proteins? Could the reactivity to PTM proteins be reduced by pre-incubation of patient serum with PTM proteins or did this only slightly reduce the reactivity of IgG with PTM proteins as described for pIgG? Did the authors perform ultracentrifugation to exclude IgG complexes that may exhibit increased reactivity to the target antigens and may occur after prolonged storage duration? Did the authors correlate the optical density results of the different anti-PTM ELISAs? If polyreactivity of IgG caused the anti-PTM reactivity, a significant correlation of the different anti-PTM reactivities should be found. We and others have shown that pIgG can be associated with false positive results even in commercially available autoantibody ELISAs for autoimmune liver diseases (4, 5).

However, there are also differences between pIgG and anti-PTM antibodies in AIH patients. Anti-PTM antibodies or at least combinations of different anti-PTM antibodies at baseline, before initiation of therapy, were associated with the treatment response in AIH patients, while we found no such association for pIgG concentrations.

Another source of bias in the detection of autoantibodies from cryopreserved serum or plasma is the increase of pIgG with storage time, as we have recently demonstrated (3). Van den Beukel et al. (1) used serum after up to 24 years of cryopreservation, a period in which we observed a relevant increase in polyreactivity of IgG. Did the authors find a correlation of reactivity in anti-PTM assays with storage time? Or did the authors normalize the reactivities in their PTM ELISAs for storage time?

Polyreactivity of IgG is not limited to autoimmune liver diseases, but is often found in diseases with high inflammation and even more often in diseases with polyclonal hypergammaglobulinemia (5). PIgG appears to be generated during class switching from IgM to IgG and during somatic hypermutation in the transition to IgG+ memory B cells (6, 7). Thus, pIgG and hypergammaglobulinemia may be produced when T helper cells stimulate B cells in a manner dependent and independent of B cell receptor specificity (6). The finding that AIH patients have an AIH-specific T cell receptor repertoire but not an AIH-specific B cell receptor repertoire is consistent with this pathophysiological concept (8). In addition, polyclonality of autoantibodies and IgG is also found in experimental mouse models of AIH (9, 10).

In conclusion, polyreactivity of IgG to multiple protein and non-protein antigens, including typical ELISA blocking reagents, is a common finding in untreated AIH. All immunoassays designed for use in patients with untreated AIH must exclude polyreactivity of IgG as a source of false positive results during the immunoassay validation process.

## Author contributions

RT: Conceptualization, Writing—original draft, Writing—review and editing. BE: Writing—original draft, Writing—review and editing. AC-M: Writing—original draft, Writing—review and editing.
